# Does childhood obesity foreshadow a future time-bomb of early and more aggressive cancers?

**DOI:** 10.3389/fendo.2025.1674961

**Published:** 2026-02-11

**Authors:** Jeff M. P. Holly, Katherine Samaras

**Affiliations:** 1 School of Translational Health Sciences, Faculty of Medicine, University of Bristol, Bristol, United Kingdom; 2 Department of Endocrinology, St. Vincent’s Hospital Sydney, Sydney, NSW, Australia; 3 St. Vincent’s Clinical School, University of New South Wales (NSW) Sydney, Sydney, NSW, Australia; 4 Clinical Obesity, Nutrition and Adipose Biology Lab, Garvan Institute of Medical Research, Sydney, NSW, Australia

**Keywords:** childhood obesity, early-onset cancer, food environment, obesity-related cancers, public health

## Abstract

There has been a global explosion in the prevalence of childhood obesity with 20% of children worldwide now growing up with excess weight and, despite many calls for interventions to redress the costs to health and society, rates continue to rise. It has also recently been observed that there is a global trend with the incidence of early-onset cancers, diagnoses prior to age 50, increasing. We outline the different lines of evidence implicating that these two trends may be linked. Conclusive proof will only be obtained when longitudinal studies, initiated after the current surge in childhood obesity, mature. This will require decades, however, due to the long time-lag between exposure and cancer presentation it would then be too late to avoid a time-bomb of early cancers. This adds considerable urgency to the calls for more effective action to prevent the current epidemic of childhood obesity. The obesity epidemic is driven by an obesogenic food system to which children are particularly vulnerable. Protecting children will require broad multisector coalitions to enable sets of mutually reinforcing policies such as front-of-pack food labelling, restrictions on the ubiquitous marketing, food taxes, subsidies and mandated healthy school meal programs.

## Introduction

Childhood obesity is arguably the greatest risk for future health globally and stands to compromise advances made in human health and life expectancy over the last century. In adults, overweight is defined as Body Mass Index (BMI: mass divided by square of height in kg/m^2^) in the range 25-29.9 kg/m^2^ and obesity as BMI exceeding 30.0 kg/m^2^. For children and adolescents aged 5–17 years, due to varying linear growth rates, classifications for overweight and obesity are based on age- and sex-specific centile cut-offs derived from averaged data from six counties and adjusted to a BMI of 25 for overweight and 30 for obesity at age 18 years, according to the criteria recommended by the International Obesity Task Force (1). Trajectories of childhood and adolescent overweight and obesity prevalence over the last quarter century raise considerable concern. A recent systematic review and meta-analysis of studies published 2000-2023 found that the global prevalence rates of overweight were, concerningly, 14.5%, with 8.5% in the obesity category (2). The Global Burden of Diseases, Injuries, and Risk Factors Study (GBD) analysis of data from 180 countries found that childhood and adolescent overweight and obesity had increased in every world region between 1990 and 2021 with a global increase in obesity of 244%. The scale of the upward rise is more concerning when considering that between the decades of 2000–2011 and 2012–2023 obesity prevalence increased by 50% (2). This means that 1 in 5 children worldwide are now growing-up with excess weight, with its physical and mental health consequences. Data from the World Obesity Federation predict that 240 million children and adolescents will live with obesity by 2025 and this figure would continue to rise to 310 million by 2030, with an additional 350 million living with overweight (3). By 2050 one third of the world’s children and adolescents (746 million) are predicted to be living with overweight and obesity with more than half in the obese classification (4). The most rapid rises are evident in northern Africa, the Middle East, Latin America and the Caribbean, however even in high-income nations children are gaining weight faster than previous generations, with obesity manifest at earlier and earlier ages. For example, of boys born in the 1960s around 7% were living with obesity by the age of 25, but this has increased to around 16% for boys born in the 1990s and is projected to reach 25% for boys born in 2015 (4). This represents a catastrophic failure of society with dire financial, economic and societal costs for the future, particularly in lower income nations that lack the health infrastructure and resources to treat the diseases caused or fueled by obesity.

Obesity is a complex chronic disease characterized by excess body fat which causes systemic physiological dysfunction and impairs health; when obesity develops in childhood it is particularly concerning due to lifelong physical and health consequences (5). Children and adolescents living with obesity face serious health issues, even before adulthood: a systematic meta-analysis reported increased risk of incident obesity-related comorbidies by youth: a 1.4-fold risk of prediabetes, a 1.7-fold risk of asthma, 4.4-fold risk of hypertension and 26.1-fold risk of metabolism-associated steatotic liver disease (5). Further, obesity in childhood tracks into adulthood: it is difficult for children who develop obesity to return to healthy weight. If obesity continues in adolescence, it rarely resolves in adulthood (5). This has considerable potential impact over the life-course on health outcomes later in life, quality of life, future economic potential, the inevitable increased adult healthcare costs and societal costs of reduced productivity and premature loss of workforce, key factors influencing a nation’s Organization for Economic Cooperation and Development status.

Over the last few years compelling evidence has emerged indicating that obesity during childhood and adolescence may also increase the risk of subsequently developing a number of cancers in early adult life (6). An earlier than expected diagnosis of cancer is particularly concerning, not just because of the younger age, but the cancers seem more aggressive, with a more advanced stage at diagnosis and worse survival outcomes compared to those with later onset obesity (7). The evidence for obesity-induced induction of early-onset cancers is at present mainly circumstantial but there are other reasons to raise alarm and if we wait for conclusive evidence, a public health crisis will be unavoidable.

Given that the concerningly high prevalence of childhood obesity has developed over the last twenty years and the incidence of most common cancers before age 30 years is very low, the detection of any immediate effects is less likely, given the blunt methods used in epidemiology with often poor covariate measures (8). However, there is accumulating evidence internationally of increasing rates of premature presentations of several cancers, that is, evident at an earlier age than previously expected (9). This is analogous to what has been observed for type 2 diabetes mellitus in children and youth, where previously this was a condition that manifested in late middle-age or older people. The annual growth in incidence rates of type 2 diabetes in children and youth in the U.S.A. for the decade 2002–2012 was 5-fold higher than that for type 1 diabetes (7.1 vs 1.4%, respectively) (10). Furthermore, the most recent global data show that almost 5-times more adolescents are living with type 2 diabetes compared to type 1 diabetes (11), an exponential rise and attributed to childhood and adolescent obesity (12). Similar to the paradigm of early onset diabetes, population studies demonstrate concerning trends that obesity in childhood may be promoting an increased risk of cancer (6).

To understand the reasons why early-onset obesity promotes carcinogenesis, it is relevant to understand recent scientific developments in how genetics and basic science have transformed our understanding of cancer pathogenesis. This new understanding provides insights into how obesity is instrumental in affecting pathways initiating or promoting cancer and why obesity exposure during childhood and adolescence may not only impact the appearance of cancers decades later, but also accelerate individual cancer susceptibility pathways.

## Paradigm shift from the mutation-centric view of cancer

**Figure 1:**
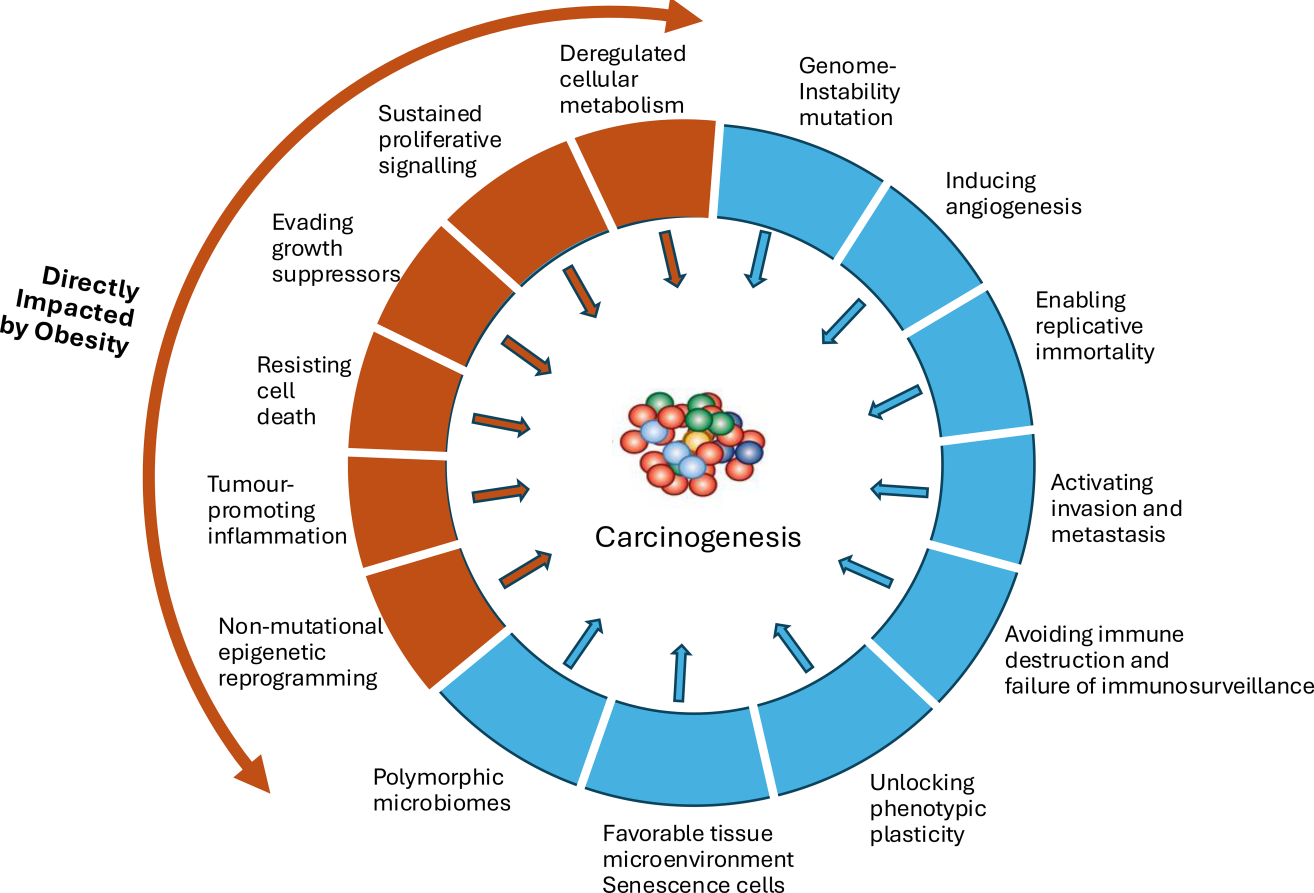
The Hallmarks of Cancer, the functional capabilities acquired by most, if not all cancers, during carcinogenesis. With an indication for those which are directly impacted by obesity.

In 1954 Armitage and Doll proposed the multi-stage theory of cancer (13). A landmark publication in 2000 then described the “Hallmarks of Cancer” which first described six key pathways that needed disruption in order for cancer to develop (14) and subsequently updated with additional pathways and other enabling characteristics (**Figure 1**) (15, 16). The paradigm was further consolidated with the “Vogelgram” multistage/sequential mutation model of carcinogenesis which suggested that sequential cell mutations disrupted each of these pathways culminating in a malignant neoplasm (17). Enthusiasm for this mutation-centric model grew as remarkable technical developments in genomics, particularly in sequencing technology, enabled the subsequent identification of many hundreds of ‘driver mutations’ in oncogenes and tumor-suppressor genes (18). However, from the start many observations contradicted this model. Back in 1949, Berenblum and Shubik proposed an alternative hypothesis of carcinogenesis suggesting “*that the carcinogen, as initiator, converts a small number of cells to latent tumor cells, and that the latter persist unchanged until promoted by a further stimulus into morphological tumors*.” (19**).** This stemmed from their observations in a mouse model of mutagen-induced cancer: mutagen exposure alone did not induce cancer, unless followed by exposure to an irritant, even if the latter was delayed for up to a year (19). This was subsequently robustly replicated in many different models and numerous other studies also indicated that acquisition of driver mutations was not the rate-limiting step in carcinogenesis (20). A further challenge to the mutagen-centric view was posed by Richard Peto who noted that as both cell size and the natural mutation rate do not vary considerably between species, but species vary considerably in size and lifespan, then larger animals with longer lifespans will involve many more cell divisions and therefore have much higher cancer rates if the risk of cancers was dependent on acquiring a carcinogenic mutations (21). That this does not occur became known as Peto’s Paradox. A human is around 3,000-fold larger than a mouse and lives 30–40 times longer which should translate to many thousand more cell divisions incurring a much greater risk of mutation-induced cancer, but evidence suggests cancer occurrence is very similar in mice and humans (22).

Basic biology indicates that DNA is not replicated with 100% accuracy introducing natural mutations: every time a cell divides mutations occur at a rate of around one per 10^9^ to 10^10^ nucleotides per cell per division (23). Cells which divide regularly throughout life, such as those within epithelial surfaces which divide every 1–4 days, will accrue thousands of mutations over decades, and everyone should therefore develop an epithelial cancer. Indeed, the evidence indicates that this does occur and in advanced adulthood we all harbor many latent cancers. Histological examination of tissues obtained at autopsy show that latent occult cancers, harboring oncogenic mutations, are present in all elderly individuals, although most had not presented with clinical disease (24, 25). This has been confirmed more recently in biopsies obtained in population cancer screening programs; for example, prostate screening studies reveal many men have *in-situ* prostate cancers that do not cause clinical disease during their lifespan (25).

Over the last two decades genetic technology advances have enabled whole genome sequencing in very small tissue biopsies. As recently reviewed, such analyses of many different tissues revealed that not only were there thousands of mutations present in tumor samples, but similar numbers of mutations were present in normal non-malignant tissues, which become patchworks of expanding clones harboring many mutations including in oncogenes and tumor-suppressor genes, as predicted by the basic biology (26). Comparative sequencing also indicated that the rate at which mutations occur in advanced cancer is similar to that in normal cells (27). With this powerful technology, major collaborations were initiated to address the ‘Grand Challenges’ of cancer (28). One of these collaborations, the Mutograph Team, set about identifying ‘mutational signatures’ for 20 known human carcinogens by undertaking whole genome sequencing of tumors induced in large numbers of mice chronically exposed to these chemical mutagens. To some surprise they found mutational signatures consistent with exposure for only 3 of the carcinogens tested, for the other 17 the pattern of mutations found in the tumors were no different from those found in tumors that spontaneously occurred in the same tissues (29). The conclusion was that the majority of these carcinogens were not actually mutagenic but just promoted the development of spontaneously initiated tumors. This also implied that spontaneously initiated cancers were present, but latent, and only developed into cancers when subjected to the promoter.

**Figure 2:**
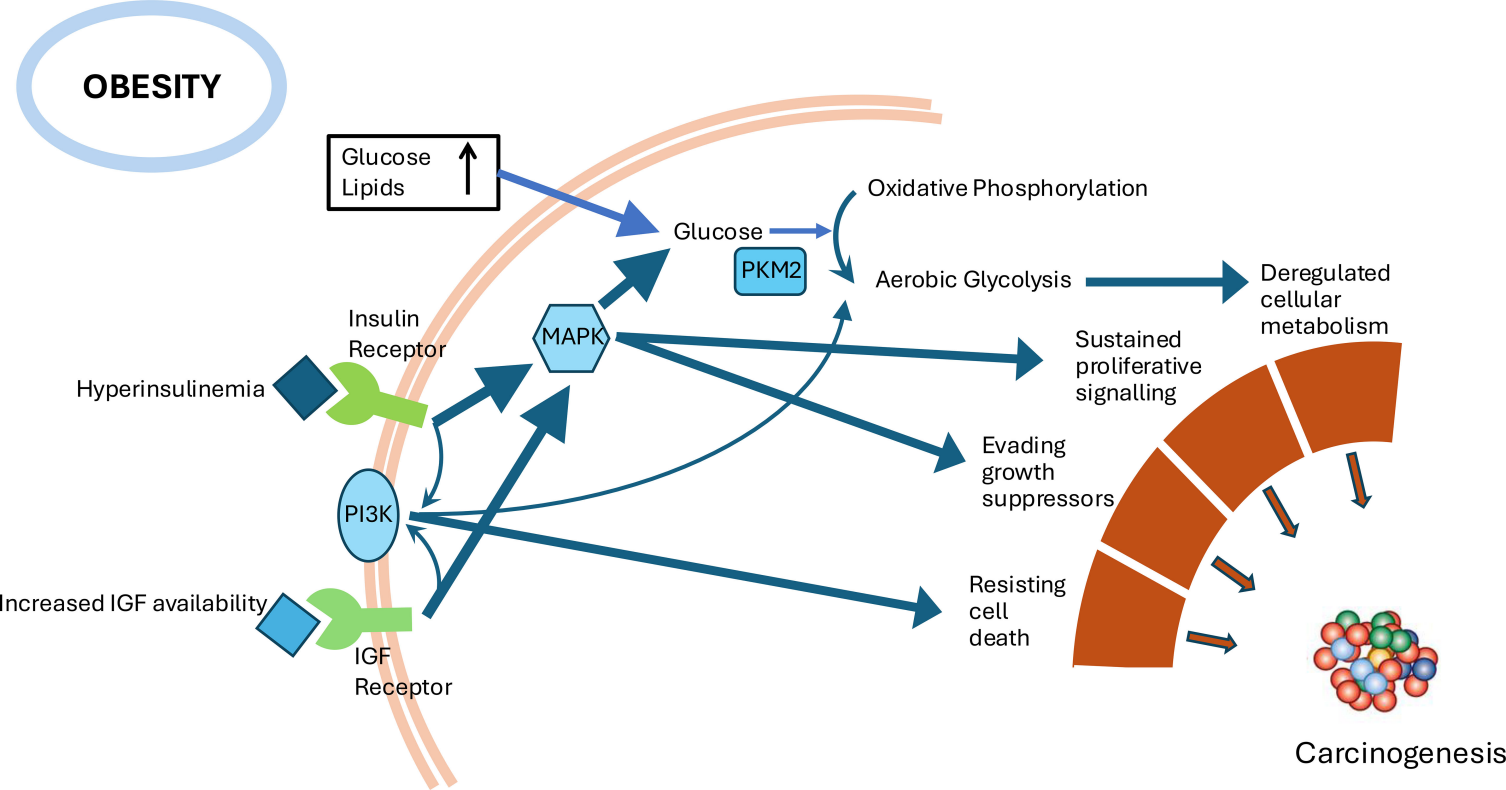
Metabolic and hormonal pathways that are perturbed in obesity which can promote the progression of carcinogenesis.

These recent observations are, consistent with the old model of an initiator creating a malignant cell that remains latent until a second exposure promotes its development into a cancer (19). This was an extension of an earlier ‘seed and soil’ hypothesis proposed by Stephen Paget in 1889 in which the ‘seeds’ were malignantly transformed cells, but which only developed into tumors if they found an appropriate ‘soil’ (30). The recent evidence indicates that over decades many cells acquire mutations in cancer driver genes and these initiation events create large numbers of ‘seeds’ but these only progress with a second promotion event: the ‘soil’ becoming suitably fertile for the seed to develop into a clinical cancer. Many of the factors associated with obesity may indeed promote the fertility of the ‘soil’ in which mutated cells may thrive (**Figure 2**). Obesity is associated with worse outcomes for established cancers; for example, obesity is associated with reduced breast cancer disease-free and overall survival, and reduced efficacy of adjuvant endocrine therapy (31). It is unknown what the impact would be if the obesity was present from childhood.

## Lifestyle and metabolism as cancer promoters

Long before mutations dominated the cancer debate it was observed that the occurrence of tumors in mice was dramatically affected by their nutritional status. As early as 1909, experiments with mouse cancer models demonstrated that caloric restriction dramatically reduced growth of transplanted tumors (32). In 1926 it was observed that excision of spontaneous tumors in fully fed mice prevented cancer recurrence in 18% of the animals; however, the addition of calorie restriction increased the rate to 73% (33). A year later the concept that metabolic status may be a critical driver of cancers was further promoted by the observation by Otto Warburg that unlike normal cells that metabolized glucose via oxidative phosphorylation, cancer cells in contrast metabolized glucose via aerobic glycolysis resulting in fermentation of the glucose to lactate (34). This enabled the metabolic fuels to not only supply energy for the cell but to also provide the precursors necessary for building the macromolecules required for the rapid cell division in cancer. It was subsequently observed that nutritional status could activate this switch in cell metabolism via the metabolic hormones insulin and insulin-like growth factors (IGFs) (35). Through their cell surface receptors, insulin and IGFs activate phosphatidylinositol-3 kinase (PI3K), directly stimulating aerobic glycolysis (36) and activating the mitogen-activated protein (MAP) kinase pathway which promotes the alternative splicing of pyruvate kinase (PK) to its less active form, PKM2, resulting in a redirection of glucose metabolic flux from pure energy generation to anabolic (i.e. cell proliferation driving) processes (37) (**Figure 2**).

The widespread adoption of commercialized Western foods with caloric excess and the associated obesity epidemic produce high flux and dysregulation through these specific metabolic pathways and may play a role in driving some cancers. The dissemination of fast or processed Western-style eating has long been implicated in the global epidemic of type 2 diabetes (38). Evidence is accumulating to indicate that it may also have consequences for cancer: for example, there are large geographic variations in cancer occurrence, with many cancers common in Western nations being relatively rare in other parts of the world (39). Whilst differences in ascertainment and genetic variations may contribute to these geographic variations, nations with previously very low cancer rates have experienced large increases in cancer prevalence in recent decades as they consume Western-style diets, contemporaneous with obesity rate increases (40). The available evidence suggests that despite large variations in clinical cancers, there are much smaller geographic variations in latent occult cancers that are only detected at autopsy suggesting that cancer-promoting, rather than cancer-initiating factors, are responsible for these variations (25). In addition, studies of human migration have consistently observed that cancer rates in migrants soon converge to the cancer rate of the population in their new locale; the rapid timeframe of such changes excludes genetic changes but rather supports environmental factors (25, 41). The convergence to local cancer rates is higher for migrants who settle in urban, versus rural areas, and increases rapidly with time following migration (42). Cumulatively, this evidence implicates that the adoption of a Western-style lifestyle with its associated metabolic disturbances, is what drives the higher rates of common cancers. Not necessarily by initiating more cancer pathways, but by promoting the naturally occurring occult cancers due to energy- and obesity-induced enhancement of key signaling pathways. (25).

## Obesity and cancer

The link between adult obesity and the presentation of age-appropriate cancers is now well established, with the International Agency for Research on Cancer (IARC) concluding that there was sufficient evidence to indicate that obesity increased the risk of cancer of the esophagus, gastric cardia, colon and rectum, liver, gallbladder, pancreas, breast (postmenopausal), endometrium, ovary, meningioma, thyroid, multiple myeloma, and renal cell carcinoma (43). An umbrella review of meta-analyses confirmed these associations (44). A recent Swedish pooled-cohort study of over 4 million people suggested a large number of additional potential obesity-related cancers could be added to those already established, including malignant melanoma in men, diffuse large B-cell lymphoma, other lymphoid cancers, myeloid cancers, oral cavity cancers, cervical adenocarcinomas, extra-hepatic bile-duct, small intestine, vulva, penis, connective tissue cancers and gastrointestinal stromal cancers (45). In 2014 of all cancers diagnosed in the USA, 40% were cancers that have been associated with overweight and obesity (46).

**Figure 3:**
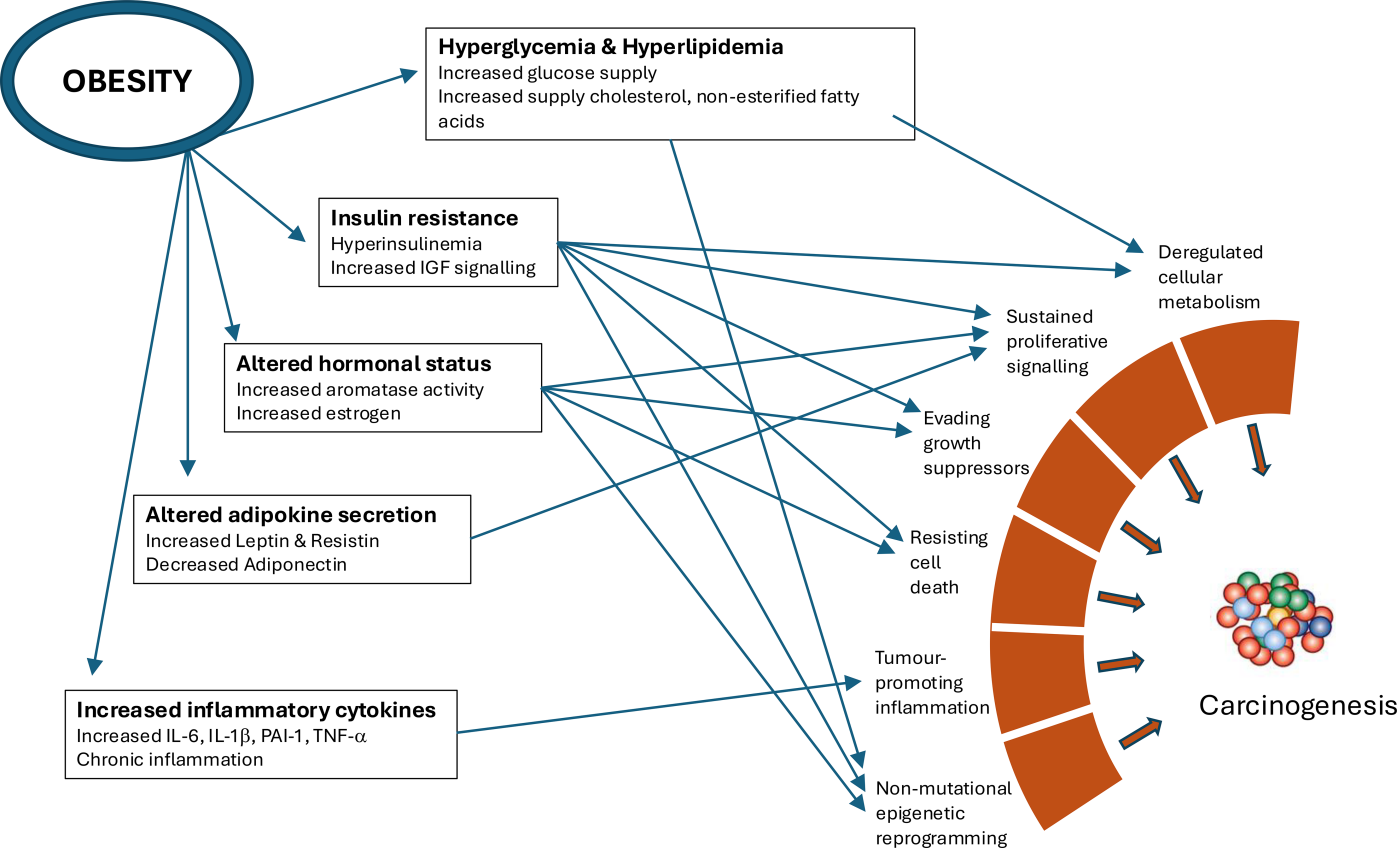
The metabolic, endocrine and immune changes that are associated with obesity and which may impact the hallmarks that drive carcinogenesis.

There are many potential mechanisms by which the metabolic, endocrine and immune changes associated with obesity may promote the hallmarks that enable cancers (**Figure 3**). These links have been the subject of many recent reviews (47–50). We will not reiterate these but will make a few remarks regarding how the evolving concepts of carcinogenesis may impact the interpretation of these links. Viewing cancer as a two-stage process involving initiation and promotion, with the latter being the rate-limiting stage, obesity is unlikely to alter the basic mutation rate or the rate of cell division in most tissues and hence will not affect the initiation stage (51). Obesity is however characterized by the altered availability and distribution of many nutrients, intermediary metabolites, microbiome dysbiosis, the levels of many hormones and a chronic low inflammatory state; all of which are known to promote the progression of cancers and have been proposed as explanations for the obesity-cancer associations (52). With the accumulation of large numbers of spontaneous mutations over decades, the ‘seeds’ with potential oncogenic driver mutations will become plentiful. The “soil’ fertility is enhanced by the milieu that characterizes obesity, thus promoting the transformation of latent occult tumors into clinical cancers (35, 47).

Several studies have observed that cancer risk increases with the duration of living with obesity. For example, in the Framingham Heart Study, every additional 2 years of living with obesity increased the risk of a cancer-related death by 3%, despite study recruitment starting in 1948 when there were relatively low obesity rates with average onset at 50 years and the average duration of living with obesity was only 13 years (53). In the longitudinal Women’s Health Initiative, which recruited women aged 50–79 years between 1993-1998, a longer duration of living with excess weight was associated with an increased incidence of all obesity-related cancers (54). Specifically, postmenopausal breast cancer risk increased 5% every 10 years living overweight and endometrial cancer risk increased 17% for every 10 years; adjusting for duration and extent of overweight increased these risks to 8% and 37%, respectively (54). These prospective studies relate to populations who predominantly became obese in their 4^th^, 5^th^ or 6^th^ decades and then developed cancers in their 6^th^, 7^th^ or 8^th^ decades (53, 54). Over the last few decades, with the onset of obesity increasingly occurring at much earlier ages in childhood and adolescence, with the likelihood of living with obesity for many decades, the consequences for incident clinical cancers may just be coming evident.

## Childhood obesity and early cancer

Early-onset cancers are defined as cancers diagnosed before age 50 years, cancers are predominantly diagnosed in people aged over 50 years. Whilst some cancers are decreasing in incidence in developed countries, partly due to reductions in cigarette smoking, an increased incidence of a number of cancers in younger adults have been observed (9). First reported from cancer registries, the most obvious explanation is that the increase was due to changes in multidomain clinical practices, including expanded cancer screening programs, incidental detection from other clinical activities, increased awareness in diagnoses introducing ascertainment bias, and even changes in disease classifications (7). To overcome such confounding many groups have employed age-period-cohort modelling to disentangle ‘age’ effects that reflect the biologic aging processes, ‘period’ effects that reflect changes in clinical practices related to ascertainment, and ‘cohort’ effects that reflect changes in risk factors and exposures unique to the year of birth (55).

Using such techniques, it has become clear that over recent decades there have been increases in the early-onset incidence of a number of different cancers above that which could be explained by detection improvements (6, 7, 56). These recent reviews report an accumulated body of evidence for increased early-onset cancers across many parts of the world that cannot be explained by age, period or cohort. Specifically, cancer increases have been observed for breast, colorectal, endometrium, esophagus, stomach, extrahepatic bile duct, gallbladder, head and neck, kidney, liver, multiple myeloma, pancreas, prostate, and thyroid. Many of these cancers are those already established as obesity-related (47, 57). In addition, the increases have paralleled and temporally followed increasing obesity prevalence. Estimates from comparative sequencing of colorectal lesions indicates that it takes around 17 years for them to develop from an early adenoma into a clinical carcinoma (27). Whether the more “fertile” tissue micro-environment, made possible through the energy excess, metabolic and hormonal disturbances of obesity, alters this time-course is yet to be determined. A study of international cancer incidence trends between 1998 and 2012 in participants aged of 15- to 39-years found the most prominent increases were in the 11 obesity-related cancers recognized by the IARC (9). A genome-wide association study (GWAS) meta-analysis and two-sample Mendelian Randomization (MR) analysis of early-onset colorectal cancer found evidence from the GWAS that the insulin-signaling pathway and immune/infection-related pathways were associated with risk of early-onset colorectal cancer, two pathways disrupted in obesity (58) (see **Figures 1**, **2**). The MR analysis revealed evidence of probable causal associations for larger body size and higher body fat percentage, waist circumference, waist-to-hip ratio, basal metabolic rate, and, importantly, fasting insulin which drives cell proliferation pathways (58).

Applying age-period-cohort modelling to analyze data from 30 cancers registered in the USA between 1995 and 2014 (14,672,409 incident cancer cases) found that for successive age cohorts born since 1950 the incidences of 6 of 12 obesity related cancers (colorectal, uterine, gallbladder, kidney, and pancreatic cancer and multiple myeloma) had increased sharply for adults younger than 50; in contrast, for 18 other cancers not known to be obesity-related, incidence in young adults only increased in two and actually decreased for around half (59). Chinese data are also informative, given the rapid upward trajectory of obesity in that nation over the past 40 years. Using cancer registry data and a similar analytical design, successive birth-cohorts from 1962 were compared, finding even stronger associations; modelling projected that the early-onset cancer incidence rate would double over the next decade (57). Chinese national survey data show that obesity was still uncommon in 1982, when the childhood prevalence of overweight and obesity (6–11 years) were 2.2% and 0.6%, respectively and for adolescents (12–17 years) just 1.0% and 0.2%, respectively (60). However, by 2015-2019, Chinese childhood overweight prevalence had tripled to 6.8% and obesity prevalence increased 6-fold to 3.6%; adolescent overweight prevalence had increased more than 10-fold to 11.1% and the obesity prevalence 16-fold to 7.9% (60). The contemporaneous changes in cancer incidence are compelling: relative to the 1962 birth cohort incidence rates, the age- and period-adjusted incidence rate for 12 obesity-related cancers had increased by 3.7-fold for those born 1982–86 and by 25.1-fold for those born 1997-2001; the increases were most evident for early-onset cancers, increasing sharply with younger age. For those aged 25–29 years the annual increase was 15.3%, with no such trend observed for those cancers not related to obesity (57).

Data derived from nations with mandatory military service are also revealing. For example, Israeli data collected at the entrance medical examination (16–19 years) since 1967 in nearly 2.3 million recruits found a gradual increase in cancer incidence across the BMI centiles at age 17-years-old (61), with a mean age at cancer diagnosis in men and women of 43.2 and 40 years, respectively. There was a 26% increase in incident cancer risk in men who were obese in adolescence and a 27% increase for women. An interesting outlier in these cohort analyses was that adolescent obesity appeared protective for breast and cervical cancers. Endocrine factors may explain this paradox: prior to pubertal ovarian maturation, adipose tissue is the major source of estrogen; high estrogen levels may result in early differentiation of mammary tissue with reduced risk of subsequent cancer (62). The increased cancer risk associated with overweight in adolescence was stronger in those recruited in later years of the study, compared to those recruited earlier in the study period, consistent with the recent marked increases in obesity (61).

The association of health-associated lifestyle scores (including maintaining a healthy weight) and composite polygenic risk scores for all-cancers and for breast cancers on the incidence of early-onset cancers was recently examined in the UK Biobank database (63). Lifestyle factors and genetic predisposition were each found to be associated with the risk of early-onset all-cancers and breast cancer, and although these associations were independent of each other, individuals with a high genetic risk appeared to have the greatest risk reduction through a healthy lifestyle (63).

The strongest evidence for a childhood obesity – early-onset cancer link comes from longitudinal life-course cohorts which include measures of adiposity and other confounders at different stages of early life, which can then be related to subsequent risk of early-onset cancers (8). A prospective observational study from Gothenburg examined the associations between BMI at ages of 8- and 20-years between 1945 and 1961 for 36,566 men; after 43-years mean follow-up overweight at age 8 was associated with a 51% increased risk of incident cancers and, for obesity-related cancers, a 38% higher cancer mortality risk; this risk was not reversed even if overweight status normalized by age 20-years (64). Other evidence indicates that maintaining overweight from childhood into adulthood cumulatively increases later incident cancer risk, consistent with evidence that obesity duration also increases cancer risk (54). Further Scandinavian registry data from Denmark quantified the risk for future gastrointestinal cancers by childhood and youth BMI. The Copenhagen School Health Records Register (CSHRR), a study of 64,695 young men examined the association between being overweight and obesity at ages 7–13 years and 17–26 years and incident gastrointestinal cancer. Childhood overweight increased the risk of subsequent esophageal and gastric cardia adenocarcinomas by 149%; this risk increased to 220% if overweight was maintained into early adulthood; however, there was little evidence of risk in those who normalized weight by early adulthood (65). A more extensive study of the same CSHRR examined the risk of obesity-related cancers in relation to childhood BMI trajectories calculated between the ages of 6 and 15 years for 301,927 children (149,325 girls) born between 1930 and 1988 (66). The incidence rate of obesity-related cancers presenting after age 30 years was greater in those with a steeper childhood BMI trajectory, with associations strongest for early-onset cancers (66). In contrast, a steeper childhood BMI trajectory was protective for subsequent pre- and postmenopausal breast cancers (66) confirming previous findings (61, 62). While providing a rich source of data, life-course cohort studies are extremely difficult and expensive to establish and maintain over extended periods. In addition, only longitudinal studies established over the last few decades would capture the large increase in childhood obesity prevalence; these will take another few decades to reveal incidences of early-onset cancers (8). Further, studies collecting data on early-onset cancers were established prior to the modern obesity epidemic in children; for example, the rate of childhood obesity in the CSHRR was just 2% (66). In addition, associations with early-onset cancers obtained from populations with slower rates of increase in childhood obesity may underestimate the potential problems facing populations which have experience much greater increases in childhood obesity. It is also possible that the factors promoting the etiology of obesity in childhood in the 1930s-1980s when overweight-obesity rates were 2% may be very different from the factors driving childhood obesity currently where rates of 30-40% have been observed in some populations.

It has been proposed that the rise in early-onset cancers was more apparent than real, based on rising incidences of 8 cancers in adults younger than 50 years in the USA not matched by mortality increases with the exception of colorectal and endometrial cancers, interpreted to be due to incidental findings and changes in diagnostic criteria (67). However, the lack of increased mortality of these serious cancers does not abrogate the impact of obesity on rising cancer rates in younger people. Rather, the lack of increased mortality likely reflects the impacts of both early detection and improvements in the treatment of these serious cancers over the past 30 years. Further, a much wider analysis of cancers across five continents compared trends in incidence and mortality for early-onset cancers with those for later-onset cancers. For early-onset cancers increased incidences were observed for 10 cancer types in females and 7 cancer types in males in at least ten countries and in many of these the increases in early-onset cancers were higher than changes in the corresponding later-onset cancers (68). Furthermore, although for some early-onset cancers there was no corresponding increase in mortality, they did observe concurrent rises in incidence and mortality for several early-onset cancers which appeared to exclude ascertainment bias (38). Changes in diagnostic and treatment regimens can seriously confound interpretation of incidence and mortality rates which remains a limitation. However, as described above, there is a consensus that obesity increases the risk of several cancer types and the available evidence indicates that this risk increases significantly with the duration of living with obesity. It would therefore be extraordinary if living with obesity from early childhood did not impact the risk of these obesity-related cancers. A more focused analysis of global incidence trends indicated that early-onset obesity-related cancers were increasing significantly faster than early-onset non-obesity-related cancers in many countries particularly in North America and Oceania (69).

## Discussion

The prevalence of obesity in childhood has increased globally at an alarming rate over recent decades with many potential life-long consequences. More recently many international reports have indicated a marked and concerning increase in the incidence of early-onset cancers, and these have mainly been cancers where the obesity association had already been established in later stages of the life span (47, 57). Emerging evidence indicates that these two trends are linked and that obesity in childhood may be a major contributing factor for the development of early-onset cancers. The time-lag between exposure and cancer presentation suggests that the rise in early-onset cancers currently being observed may be the tip of an iceberg, only reflecting the earliest stages of the current childhood obesity epidemic (6, 47). If this is the case, then the recent large surge in childhood obesity may foreshadow a future public health time-bomb of early-onset cancers (6). More conclusive proof of the link may require life-course population studies that have recruited children since the high rates of childhood obesity have occurred and for such cohorts to mature to ages that allow assessment of incident cancers before the age of 50 years. This may take several more decades, by which time it would be far too late to respond with any preventative measures that could alter the course and prevent an epidemic of early cancers for several future generations. At the very least there needs to be increased awareness of the potential threat among the public health, primary health, epidemiology, pediatric and endocrinology communities.

International bodies such as the Global Burden of Diseases, Injuries, and Risk Factors Study Adolescent BMI Collaborators have called for immediate actions to address the impending public health crisis (4). This was without accounting for the potential time-bomb of early-onset cancers that could result from childhood obesity which would add enormous health, social and economic costs to society. Attempts to address childhood obesity have, to date, predominantly relied on lifestyle-based, individual-orientated behavior-change strategies (70). The continued rise in obesity rates to epidemic proportions indicate that such strategies have failed and that governmental actions are required to directly address the drivers of obesity at a population-level. A cultural shift is required to change the obesogenic environment in which children grow up, particularly their food environment and exposures to food marketing. This concurs with the World Health Organization’s urgent call for action on childhood obesity (71).

**Figure 4:**
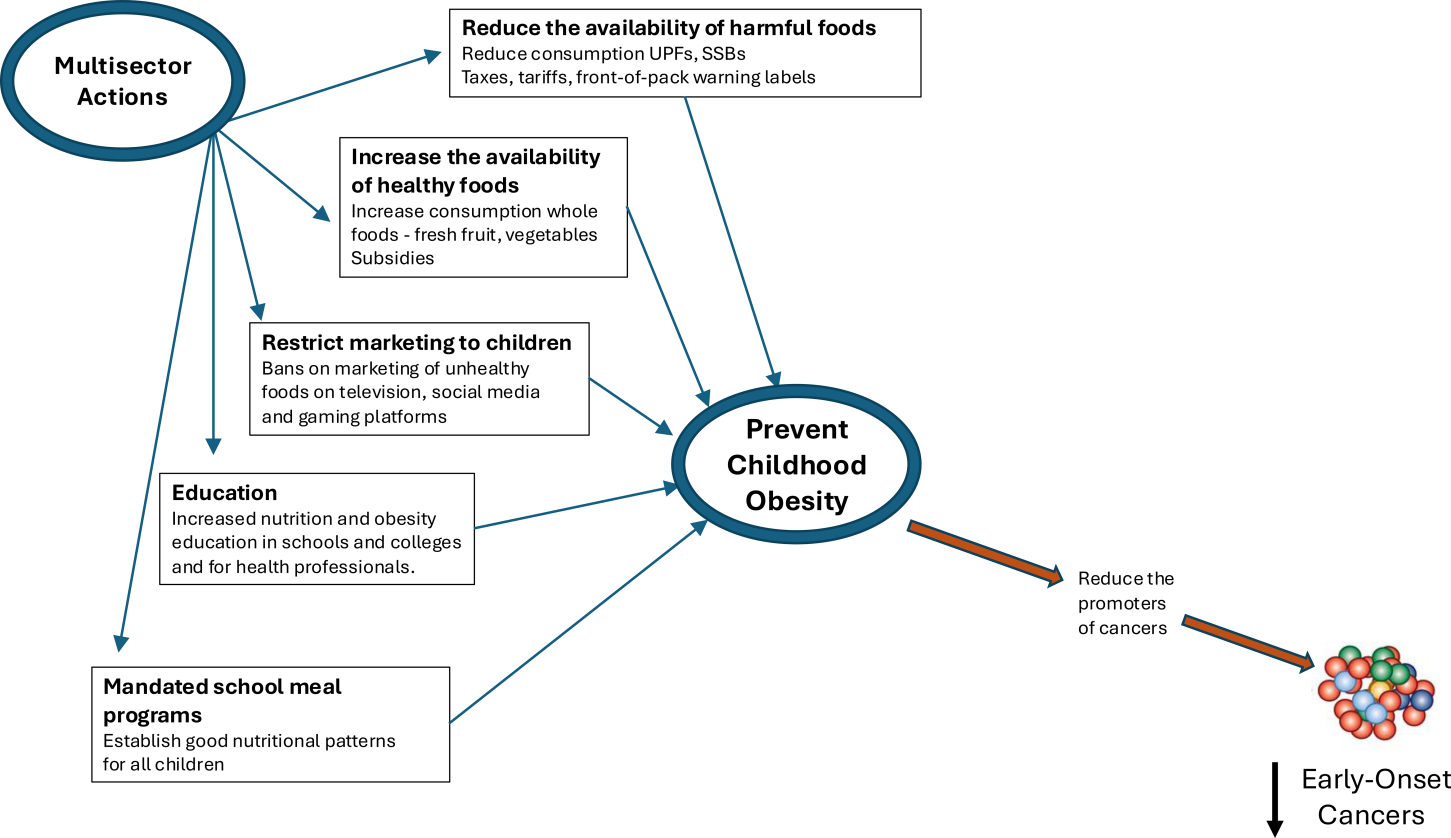
Multisector, political and societal, actions required to invoke the evidence-based mutually reinforcing policies that can reduce the prevalence of childhood obesity and prevent the potential development of subsequent early-onset cancers.

The potential that obesity in childhood may increase the risk of early onset cancers adds to the already clear health challenges that they may face throughout life and strengthens the need for urgent action. There have been several recent extensive proposals of frameworks describing integrated strategies to address the growing obesity epidemic, emphasizing the need for stronger government actions that mandate changes to the food environment (72–74). This will require overcoming the economic and political power of the food industry that has successfully obstructed, weakened or delayed prior government actions as outlined in the recent report ‘Feeding Profit: How food environments are failing children’ from the United Nations Children’s Fund (75). The current food system not only drives the obesity epidemic but is also a major contributor to the climate crisis (74). Co-framing the parallel and overlapping threats and common solutions involved in reducing obesity, protecting the environment, and avoiding climate breakdown should help to build the coalitions across society that will be required to overcome the obstacles and convince governments to mandate effective policies (74, 76). Broad multisector coalitions will help foster the required sets of mutually reinforcing policies such as front-of-pack food labelling, marketing restrictions, food taxes, subsidies and public procurement of healthy foods (73, 74) (**Figure 4**). Children in particular need protection as childhood is a period of physiological and emotional development with persisting organ and neurological plasticity and obesity during this period may have lasting consequences (77). Marketing to children is also of particular concern since they are vulnerable being highly impressionable, motivated by immediate gratification, and have underdeveloped nutritional knowledge and food choices made at this critical stage of development can set the foundation for a healthy life (78, 79). Protecting children from ubiquitous exposure to commercial influences requires bold actions such as the recent restriction of social media access to children in Australia (80).

Education is of foundational importance to the success of public health measures to improve diet and lifestyle. More education regarding nutrition and obesity is needed in schools and colleges. In addition, evidence indicates medical professionals are inadequately educated in nutrition and obesity to treat patients and tackle the obesity epidemic and much more emphasis on these issues is needed in medical schools and for the training of all healthcare professionals (74).

Mandated school meal programs can help establish good nutritional patterns. An exemplar is the National School Feeding Program that has been adopted throughout Brazil, this mandates that schools procure foods that are mostly unprocessed or minimally processed with a maximum of 10% processed or UPF and 30% to be obtained from local farms which has increased the proportion of fruit, vegetables and legumes in school meals (81–83). Another innovative approach has been taken in Vietnam with a program promoting the use of school gardens to produce fresh foods for the children (84).

Political commitment with a long-term view will be required to bring about these multisector actions to enable transformation of current childhood and adolescent diets and confront the current commercial influences. Reducing childhood obesity requires concerted and committed efforts from clinicians and allied health professionals at the coal face of clinical medicine, commitment from health service administrators to allocate workforce, policymakers to implement preventative strategies that are already known, industry to cohesively create products with consumer health as one of their authentic aims and as a successful marketing strategy. Agile industry leaders embracing these approaches will likely have flow on effects through multiple industries, since consumer uptake can be expected to be high and profitable. Clearly, a multi-pronged approach is required.

## Data Availability

The original contributions presented in the study are included in the article/supplementary material. Further inquiries can be directed to the corresponding author.
